# The Effect of Yeast and Roughage Concentrate Ratio on Ruminal pH and Protozoal Population in Thai Native Beef Cattle

**DOI:** 10.3390/ani12010053

**Published:** 2021-12-28

**Authors:** Kampanat Phesatcha, Burarat Phesatcha, Metha Wanapat, Anusorn Cherdthong

**Affiliations:** 1 Department of Animal Science, Faculty of Agriculture and Technology, Nakhon Phanom University, Nakhon Phanom 48000, Thailand; 2 Department of Agricultural Technology and Environment, Faculty of Sciences and Liberal Arts, Rajamangala University of Technology Isan, Nakhon Ratchasima 30000, Thailand; Burarat_kat@hotmail.co.th; 3 Tropical Feed Resources Research and Development Center (TROFREC), Department of Animal Science, Faculty of Agriculture, Khon Kaen University, Khon Kaen 40002, Thailand; metha@kku.ac.th

**Keywords:** beef cattle, yeast addition, rumen fermentation, feed digestibility

## Abstract

**Simple Summary:**

As a result of the recent ban on antibiotics in feed, animal probiotics are becoming increasingly popular. Yeast is extensively used as both a probiotic and prebiotic in the gastrointestinal tracts of ruminants. The purpose of this study is to determine how adding yeast (*Saccharomyces cerevisiae*) to the diet and changing the roughage-to-concentrate ratio (R:C ratio) affects nutrient consumption, rumen fermentation, microbial protein synthesis, and protozoal population in Thai native beef cattle. The roughage source was urea–calcium-hydroxide-treated rice straw. The findings suggest that supplementing with a R:C ratio of 40:60 and a LY of 4 g/hd/d boosted nutrient digestibility, volatile fatty acid (VFA) production, propionic acid (C_3_) in particular, and microbial protein synthesis while lowering protozoal population.

**Abstract:**

The objective of this research is to investigate the effect of yeast (*Saccharomyces cerevisiae*) adding and roughage-to-concentrate ratio (R:C ratio) on nutrients utilization, rumen fermentation efficiency, microbial protein synthesis, and protozoal population in Thai native beef cattle. Four Thai native beef cattle, weighing an average of 120 ± 10 kg live weight, were randomly assigned to four dietary treatments using a 2 × 2 factorial arrangement in a 4 × 4 Latin square design. Factor A was the level of roughage-to-concentrate ratio (R:C ratio) at 60:40 and 40:60; factor B was the levels of live yeast (LY) supplementation at 0 and 4 g/hd/d; urea–calcium-hydroxide-treated rice straw were used as a roughage source. Findings revealed that total intake and digestibility of dry matter (DM), organic matter (OM), and crude protein (CP) were increased (*p* < 0.05) by both factors, being greater for steers fed a R:C ratio of 40:60 supplemented with 4 g LY/hd/d. Ruminal ammonia nitrogen, total volatile fatty acid (VFA), and propionate (C_3_) were increased (*p* < 0.05) at the R:C ratio of 40:60 with LY supplementation at 4 g/hd/d, whereas rumen acetate (C_2_) and the C_2_ to C_3_ ratio were decreased (*p* < 0.05). With a high level of concentrate, LY addition increased total bacterial direct counts and fungal zoospores (*p* < 0.05), but decreased protozoal populations (*p* < 0.05). High-concentrate diet and LY supplementation increased nitrogen absorption and the efficiency of microbial nitrogen protein production. In conclusion, feeding beef cattle with 4 g/hd/d LY at a R:C ratio of 40:60 increased C_3_ and nutritional digestibility while lowering protozoal population.

## 1. Introduction

One of the approaches used to enhance growth performance of beef cattle is the use of a high-energy diet. High-energy diets are defined by a high carbohydrate content, which can lead to rapid ruminal production of short-chain fatty acids (SCFAs), which are the ruminant host’s principal energy source [1]. The fast accumulation of SCFAs in the rumen reduces buffering capacity and may result in subacute ruminal acidosis (SARA) [2,3].

It has been reported the LY can reduce rumen pH fluctuation, in part through decreasing lactate accumulation in the rumen fluid due to activation of microbes which change lactate into SCFAs [4]. When LY is added to a meal that is high in rapidly fermentable carbohydrates, the lactate content drops and the rumen pH rises. It may be higher in starch-rich meals because starch-rich diets result in a larger acid load, requiring more physically effective fiber to maintain a normal rumen environment [5]. The more stable rumen environment caused by introducing LY to starch-rich diets may result in less demand for physically effective fiber, justifying improvements in dry matter intake that improve milk and milk component production [6]. Rumen acidosis can occur as a result of carbohydrate formulation errors in diets that are already high in starch [7]. LY was primarily used in young ruminants to promote gut health and to promote the formation of the intestinal bacteria involved in feed digestion. Further developments resulted in more sophisticated LY combinations aimed at increasing fiber digestion and reducing ruminal acidosis in mature cattle [8].

LY has been found to help ruminants digest feed as well as increasing digestibility of nutrients, enhancing VFA proportions, minimizing pH decreased, lowering ruminal ammonia nitrogen, and improving microbiome [9,10]. So far, LY has been shown to give rumen microbes numerous growth factors, provitamins, and other stimulants. LY lowers the rumen’s redox potential and encourages the proliferation of bacteria—mostly cellulose consumers—which increases the rate of fiber decomposition [11]. Additionally, *S. cerevisiae* can scavenge the available oxygen to maintain metabolic activity, lowering the rumen’s redox potential [12].

Broadway et al. [13] found that yeast supplementation had a variety of impacts in the rumen, including higher ruminal pH and VFA concentrations, decreased methane (CH_4_) generation, and increased overall numbers of microorganisms and cellulolytic bacteria. A reduction of enteric CH_4_ production has been noted in cattle supplemented with 4 g of *S. cerevisiae*. Thus, LY can be another feed strategy for improving environmental circumstances [14]. As a result, the goals of this study were to investigate the impact of the R:C ratio with LY addition on total feed intake, feed digestibility, rumen characteristics and microbial population in Thai native beef cattle.

## 2. Materials and Methods

### 2.1. Ethical Procedure

Animal involved in this study were approved by the Animal Ethics Committee of Nakhon Phanom University (AENPU A2/2560), based on the Ethics of Animal Experimentation of National Research Council of Thailand.

### 2.2. Dietary Treatments and Experimental Design

Four Thai native beef cattle, weighing an average of 120 ± 10 kg live weight, were randomly assigned to four dietary treatments using a 2 × 2 factorial arrangement in a 4 × 4 Latin square design. Factor A was the level of roughage-to-concentrate ratio (R:C ratio) at 60:40 and 40:60; factor B was the levels of live yeast (LY) supplementation at 0 and 4 g/hd/d (6.0 × 10^6^ colony-forming units); urea–calcium-hydroxide-treated rice straw (*Oryza sativa* L.) was used as a roughage source. Table 1 shows the diet compositions and ingredients of concentrate mixtures, and urea-calcium-hydroxide-treated rice straw. Before the treatments were imposed, the animals were injected with 10 mL of vitamins A, D_3_, and E and were drenched with anthelmintics prior to the experiment. Two kilograms of urea and of calcium hydroxide (Ca(OH)_2_) were added to 100 litters of rice straw to make the rice-straw-treated urea and (Ca(OH)_2_) [15]. The brewer’s yeast strain *S. cerevisiae* (6 × 10^6^ cfu/g, Renu Nakhon, Nakhon Phanom, Thailand) was collected from the Renu Nakhon district, Nakhon Phanom Province, Thailand.

The experiment was conducted for 4 periods of 21 days each, with 14 days for treatment adaptation and feed intake measurements, and the last 7 days were for sample collection of feeds, fecal, and urine. The animals were fed ad libitum with water and mineral blocks available at all times (each kg of mineral block contains the following: Vitamin A—10,000,000 IU; Vitamin E—70,000 IU; Vitamin D—1,600,000 IU; Fe—50 g; Zn—40 g; Mn—40 g; Co—0.1 g; Cu—10 g; Se—0.1 g; I—0.5 g).

### 2.3. Sample Collection and Chemical Analyses

Feeds and refusals were collected daily during the experimental period and were composited by period prior to chemical analysis. Feeds, fecal, and urine samples were collected during the last seven days of each period. Fecal samples were collected by rectal sampling, the urination of each animal was collected by manual stimulation of the penis. Composited samples were oven-dried and ground (1 mm screen using a Cyclotech Mill, Tecator, 1093, Hoganes, Sweden) and then analyzed for DM, ash, and CP content [16], and acid-insoluble ash (AIA). AIA was used as an indicator to estimate DM fecal excretion [17]. Fiber fractions (NDF and ADF) were analyzed according to Van Soest et al. [18]. Metabolizable energy (ME) was calculated according to Robinson et al. [19]. Digestible organic matter fermented in the rumen (DOMR) was calculated according to an ARC [20] as follows: DOMR (kg/d) = digestible organic matter intake (DOMI, kg/d) × 0.65, where DOMI = [digestibility of organic matter (kg/kg DM) × organic matter intake (kg/d)]/100, 1 kg DOMI = 15.9 MJ ME/kg [21].

At the last day of each period, rumen fluid sample and blood were collected at 0 and 4 h post morning feeding. At each sampling time, approximately 200 mL of rumen content was collected by an oral-gastric tube (RFE, Drench-Mate, Washington, DC, USA) connected with a vacuum pump (Solida M730, J. Summit Co. Ltd., Bangkok, Thailand) at each time. Rumen fluid was measured for pH and temperature immediately (Hanna Instrument HI 8424 Microcomputer, San Francisco, CA, USA). Rumen fluid samples were filtered through four layers of cheesecloth. The first part was used to determine ammonia nitrogen (NH_3_-N) using the micro-Kjeldahl methods [16]. Rumen fluid samples were used for VFAs analysis using high-performance liquid chromatography (HPLC; Model Water 600; UV detector, Millipore Corp. Milford, MA, USA) [22]. The second part of the filtered fluid sample was fixed using a solution of 10% formalin in sterilized 0.9% saline. The total direct count of bacteria, protozoa, and fungi was obtained using the method of Galyean [23].

A blood sample (about 10 mL) was collected from a jugular vein into tubes with 12 mg of EDTA. Plasma was obtained by centrifuging at 500× *g* for 10 min (Table Top Centrifuge PLC-02, Tustin, CA, USA) and stored at −20 °C for later blood urea nitrogen (BUN) [24]. Urine samples were collected to be analyzed for allantoin by HPLC. Samples were then analyzed for urinary allantoin and creatinine. The number of microbial purines absorbed was calculated from purine derivative excretion as described by Chen and Gomes [25]. Microbial crude protein (MCP) (g/d) = 3.99 × 0.856 × mmol of purine derivatives were excreted following the method of Galo et al. [21]. 

### 2.4. Statistical Analysis

Data were analyzed according to a 4 × 4 Latin square design with 2 × 2 factorial arrangements of treatments using the General Linear Models procedures [26]. 

The MIXED procedure [26] was used to analyze data of rumen fluid parameters at different feeding times as repeated measures over time using the following model:

Y_*ijk*_ = µ + D_*i*_ + A_*j*_ + P_*ij*_ + H_*k*_ + (DT)_*jk*_ + ε_*ijk*_,

where Y_*ijk*_ is the observation from animal *j*, receiving diet *i*, in period *k*; µ—the overall mean; D*_i_*—effect of treatment (*i* = 1–4); A*_j_*—the effect of animal (*j* = 1–4); P*_k_*—the effect of period (*k* = 1–4); H*_k_*—the effect of time after feeding (*k* = 1 and 4); (DT)*_jk_*—the interaction of treatment × time after feeding; *e_ijk_*—the residual effect.

Treatment means were compared by Tukey’s multiple comparison test. Differences between means with *p* < 0.05 were accepted as representing statistically significant differences.

## 3. Results and Discussion

### 3.1. Dry Matter Intake and Nutrients Digestibility

There was no significant interaction effect between the R:C ratio and LY addition on nutrient digestibility. Moreover, DM intake was found to have significant interaction with both the R:C ratio and LY addition (Table 2). Increasing the concentrate proportion increased (*p* < 0.05) DM intake and digestibility of DM, OM, and CP but decreased (*p* < 0.05) NDF and ADF digestibility. These findings agree with Chen et al. [27], cow fed a high concentrate diet showed significantly increased digestibility of nutrients. It could be due to the concentrate diet can provide additional nutrients for rumen microbial growth and can enhance rumen fermentation. The increase in DM and OM due to greater concentrate proportion in the diet is a direct reflex of the greater digestibility of ingredients that conform to the concentrate compared with the digestibility of treated straw [15]. The lower digestibility for fiber fraction (NDF and ADF) could be due to the tendency of a lower ruminal pH to greater quantities of soluble carbohydrates in these treatments (Table 3). Some strains of *S. cerevisiae* have been shown to enhance rumen bacterial growth and activity with a subsequent effect on NDF digestibility. Moreover, LY increases fibrolytic fungi population, which have been shown to stimulate the breakdown of polysaccharides connected with lignin via esterase secretion [10,28].

It has been proposed that consumption can be lowered in diets with increased R:C ratios due to the negative relationship between forage NDF and diet bulk density [5,29]. However, increasing the proportion of concentrate in the diet had an influence on DM intake, according to the current findings.

The DM intake and digestibility of DM, OM, CP, NDF, and ADF increased with the addition of LY. Maximum digestibility of NDF and ADF was achieved at the R:C ratio of 40:60 and the addition of LY at 4 g/hd/d. Similarly, Guedes et al. [30] stated that the addition of LY improved the fiber degradation of maize silage in cows. Mir and Mir [31] found that adding *S. cerevisiae* enhanced the digestibility of DM. Furthermore, adding dry LY to growing and finishing beef calves at a rate of 10 g/d improved DM and NDF digestibility [32]. The addition of *S. cerevisiae* improved nutrient digestibility, and this could be attributed to the rumen microbe being stimulated to a greater rate of feed digestion. Furthermore, *S. cerevisiae* reduces fluctuations in ruminal pH and redox potential in high proportion of concentrate diet, resulting in more stable rumen environment for fibrolytic bacteria growth [33]. Resulted in greater amount of fiber digested, it indicates that LY addition increased both rates of fiber digestion and passage, resulting for higher intake and total digestibility [32,34].

A less acidic, more anaerobic ruminal environment might aid fiber-degrading microorganism proliferation and increase fiber decomposition in the rumen [4]. The addition of LY improved OM digestibility, and the advantages increased as the dietary NDF concentration increased. LY is hypothesized to improve animal performance by altering the rumen microbiota to promote the growth and activity of fibrolytic microbes as well as those that metabolize lactate. This could be useful in diets that encourage enhanced short-chain fatty acid synthesis in the rumen, resulting in lower rumen pH. Dias et al. [35] discovered that supplementing with *S. cerevisiae* reduced the rate of starch digestion in the rumen in cows with higher DM consumption, which could result in a more stable rumen ecology.

### 3.2. Ruminal Parameters and Blood Metabolite

An interaction effect was not found between the R:C ratio and the LY addition on ruminal parameters and BUN concentrations (Table 3). All treatments had pH levels ranging from 6.3 to 6.6. However, the results showed that as the number of concentrates in the diet increased, ruminal pH declined, and it was lowest when the R:C ratio was 40:60. Ruminal pH was found to be steady between 6.3 and 6.6, and ruminal temperature was found to be between 38.8 and 39.3 °C. However, Ramos et al. [36] discovered that diets high in concentrate typically resulted in a significant decrease in ruminal pH, a decreased digestion rate, and decreased cellulolytic bacteria activity. Ruminal pH and its daily fluctuations can be considered a major factor in the occurrence of SARA and the regulation of microbial activity. Ruminal pH is a good indicator of the rumen environment’s internal homeostasis; thus, maintaining a steady stable ruminal pH is critical for good rumen ecology, fermentation, and microbial development. In general, lower lactic acid levels in the rumen are associated with pH stabilization. Moreover, an increase in ruminal pH could be caused by a decrease in lactic acid concentration due to ability of LY to enhance lactic-acid-utilizing bacteria (*Selenomonas ruminantium* and *Megasphaera elsdenii*) [4,11,32]. Furthermore, the addition of LY enhanced ruminal pH and cellulolytic bacteria [6,30,35].

Ammonia is the primary nitrogen source for microbial protein synthesis, and bacteria can grow using NH_3_-N as the main nitrogen source. The NH_3_-N content in the current study ranged from 12.3 to 16.9 mg/dL. In the R:C ratio of 40:60, the NH_3_-N was greatest. This could be because a greater CP from a high-concentrate feed ratio may provide more microbial degradation into high concentrations of NH_3_-N than a low-ratio concentrate diet. A concentration of NH_3_-N in the range of 15 to 30 mg/dL may improve voluntary feed intake, microbial protein synthesis, nutrient digestibility, and rumen ecology, whereas an NH_3_-N deficit decreases the bacterial growth rate [37,38,39]. The greater concentration of ruminal NH_3_-N in the high-concentrate diet was attributed to the dynamic equilibrium between NH_3_-N production and use by rumen bacteria. The presence of high levels of NH_3_-N in the rumen shows that animals may be receiving adequate accessible nitrogen content from dietary intake, most likely as a result of the high concentrate content of the cow diet [36]. However, the addition of LY significantly enhanced the NH_3_-N content (*p* < 0.05), contrary to the findings of Li et al. [33], who showed that the addition of *S. cerevisiae* had no effect on the NH_3_-N concentration in the rumen. According to Kumprechtova et al. [39], lower NH_3_-N concentrations could be due to increased cellulolytic bacteria number, as this type of bacteria primarily uses NH_3_-N as a N source. The drop in ruminal NH_3_-N content appeared to be related to increased NH_3_-N absorption into microbial proteins, most likely as a result of *S. cerevisiae* stimulating microbial activity [40]. The decrease in LY supplied to cattle rumen appears to be related to NH_3_-N incorporation into microbial protein [41], or it could be due to LY’s inhibitory influence on proteolysis [42,43].

On total VFA and VFA profiles, there was no interaction effect between the R:C ratio and LY addition (Table 3). The total VFA concentrations in all treatments varied from 92.4 to 105.0 mmol/L, which was consistent with the findings of Cagle et al. [32] and Kumprechtova et al. [39]. The increased amount of concentrate in diet raised total VFA and C_3_, while decreasing C_2_ and C_2_-to-C_3_ ratios (*p* < 0.05). This could be owing to the presence of highly degradable carbohydrates, particularly starch, in the concentrate.

Dias et al. [35] discovered that dairy cows fed *S. cerevisiae* had greater total VFA and C_3_ concentrations. The addition of *S. cerevisiae* to the rumen also changed the molar proportion of VFA in the rumen—particularly, the C_3_ concentration—resulting in an increase in the ruminant’s glucogenic potential. Wang et al. [38] examined the addition of *S. cerevisiae* to in vitro gas fermentation and found no change in the C_2_ to C_3_ ratio. This difference may be impacted by different yeast strains and the types of diets utilized in various tests [9]. The addition of LY to the rumen may have enhanced the microbial population in the rumen, resulting in better carbohydrate fermentation into VFAs. The ability of LY in the rumen could aid in the formation of lactate-consuming and cellulolytic bacterial populations, hence aiding in rumen stabilization and boosting the rumen’s capability to digest fiber [10,44]. Dietary composition has the greatest influence on volatile fatty acid content; manipulating the diet by increasing the level of concentrate diet is a strategy to improve ruminal fermentation by increasing propionogenesis [45]. Such a typical propionic acid fermentation reaction was found in the current investigation, and as a result, the C_2_:C_3_ ratio decreased, which is consistent with prior findings [4,46,47,48]. This increase in C_3_ concentration can be attributed to increased nonfiber carbohydrate consumption.

### 3.3. Microbial Population and Microbial Protein Synthesis

In the current study, there were no interactions between the R:C ratio and the addition of LY on microbial population (Table 4). Total bacterial direct counts and fungal zoospores were increased (*p* < 0.05) in a high-concentrate diet, whereas protozoal populations were not changed (*p* > 0.05). The changes in the rumen microbial population are expected to stimulate the digestion of carbohydrates in the rumen, which likely explains the increased rumen NDF digestion in the present experiment and the digestion of OM. This is in agreement with Histrov et al. [49], reported that there was more carbohydrate for microbial fermentation and incorporation of NH_3_-N into microbial cells with increased feed intake. However, Ramos et al. [36] reported that the high-concentrate diet reduced the richness and diversity of the rumen microbiota. The total population of protozoa in ruminal fluid normally enhances with the high proportion of concentrate diet [3,50].

According to the findings, the population of total bacteria and fungi increased (*p* < 0.05) while protozoal decreased (*p* < 0.05) with LY addition. It could be because LY supplementation may give growth factors such as organic acid or vitamins, which may boost the population of cellulolytic bacteria. Li et al. [33] revealed that the addition of LY to dairy cows has increased the relative abundance of microorganisms used for cellulolytic, amylolytic, and lactate use. 

The results of the present study showed that microbial nitrogen supply (MNS) and efficiency of microbial nitrogen synthesis (EMNS) were increased in cattle receiving the greater concentrate proportion and with LY supplementation (*p* < 0.05) (Table 5). Rumen NH_3_-N from protein degradation would be incorporated for microbial protein synthesis. Nitrogen absorption and retention are good indicators of ruminant utilization [25]. Dias et al. [35] showed that rumen microbial protein synthesis was an effective indicator of nitrogen utilization. Furthermore, a close relationship between the protein and the carbohydrate is essential for effective utilization. However, in this study, LY supplementation significantly improved the N absorption, N retention, and EMNS. Moreover, Erasmus et al. [43] observed an increase in the efficiency of the synthesis of microbial proteins in response to *S. cerevisiae* addition. When the cows were fed LY, especially when mixed with the high-concentrate diet, this was linked to lactate utilizing bacteria, an increase in cellulolytic bacteria, and flow of MNS from the rumen. Cows fed a high-nonfiber-carbohydrate diet had increased microbial protein yield when supplemented with *S. cerevisiae*, as reported by Hristov et al. [49]. Furthermore, Dias et al. [35] discovered that cows fed high starch and LY supplementation had the highest microbial N output, and that the concentration of NH_3_-N in dairy cows fed diet with the high-starch content was lower than cows provided the low starch diet.

Furthermore, the presence of LY in the rumen increases microbial growth, resulting in increased microbial protein transport to the duodenum. In general, yeast supplementation modifies the rumen microbiome population, resulting in improved VFA production and greater microbial protein synthesis [42,46,50]. When cows were fed *S. cerevisiae* at a rate of 56 g/hd/d, microbial protein synthesis increased by 9.3 percent [49]. A decrease in protozoal populations in response to *S. cerevisiae* fermentation product supplementation, according to Zhu et al. [46], may reduce bacterial preying and allow more microbial protein to reach the small intestine. It has been found that *S. cerevisiae* supplementation reduced the amount of Entodinium in steers 26.0%. Entodinium can engulf bacteria and reduce the passage of microbial protein to the small intestine [51,52]. 

## 4. Conclusions and Recommendations

In conclusion, in Thai native beef cattle fed urea–calcium-hydroxide-treated rice straw as a roughage source, a R:C ratio of 40:60 and LY of 4 g/hd/d resulted in controlled ruminal pH, increased nutrient digestibility of DM, OM, CP, and ruminal VFA production—particularly, C_3_—and EMNS while decreasing protozoal population.

## Figures and Tables

**Table 1:** Ingredient composition of concentrate and chemical composition of concentrate and treated rice straw offered to steers.

Items	Concentrate	Urea and Calcium Hydroxide Treated Rice Straw
Ingredients, % as fresh basis		
Cassava chip	75.0	
Coconut meal	15.0	
Rice bran	4.0	
Urea	2.5	
Molasses	2.0	
Mineral mixture	0.5	
Salt	0.5	
Sulfur	0.5	
Total	100.0	
Chemical composition		
Dry matter, %	92.7	55.1
% of dry matter		
Organic matter	92.6	88.7
Ash	7.4	11.3
Crude protein	14.1	5.6
Neutral detergent fiber	27.2	72.8
Acid detergent fiber	13.4	45.9

**Table 2:** Effect of roughage-to-concentrate ratio and live yeast supplementation on voluntary feed intake and nutrient digestibility in Thai native beef cattle.

Items	R:C at 60:40		R:C at 40:60		SEM	Interaction		
	LY 0	LY 4	LY 0	LY 4		R:C	LY	R:C × LY
Dry matter intake								
kg/d	2.5	3.1	2.6	3.3	0.09	0.048	0.036	0.042
g/kg BW0.75	83.7	85.8	84.3	88.6	1.24	0.050	0.041	0.051
Estimate energy intake								
ME, MJ/d	25.6	33.1	27.3	36.5	0.82	0.042	0.039	0.971
ME, MJ/kgDM	10.2	10.7	10.5	11.1	0.98	0.024	0.031	0.852
Nutrient digestibility, %								
Dry matter	61.5	66.4	64.8	68.5	0.01	0.048	0.040	0.635
Organic matter	64.4	67.1	66.1	69.6	0.04	0.021	0.035	0.664
Crude protein	64.5	66.8	67.5	69.4	0.03	0.029	0.042	0.814
Neutral detergent fiber	60.1	66.2	58.3	63.7	0.04	0.047	0.015	0.385
Acid detergent fiber	51.6	55.2	44.1	47.6	0.05	0.030	0.022	0.638

R:C ratio = roughage-to-concentrate ratio; LY 0 = unsupplementation of live yeast; LY 4 = supplementation live yeast at 4 g/hd/d; SEM = standard error of the mean. Metabolizable energy (ME) was calculated according to the equation described by Robinson et al. [19]. Digestible organic matter fermented in the rumen (DOMR) was calculated according to the equation described by ARC [20] as follows: DOMR (kg/d) = digestible organic matter intake (DOMI, kg/d) × 0.65, where DOMI = [digestibility of organic matter (kg/kg DM) × organic matter intake (kg/d)]/100, 1 kg DOMI = 15.9 MJ ME/kg [21].

**Table 3:** Effect of concentrate level and live yeast supplementation on fermentation characteristics and blood urea nitrogen in Thai native beef cattle.

Items	R:C at 60:40		R:C at 40:60		SEM	Interaction		
	LY 0	LY 4	LY 0	LY 4		R:C	LY	R:C × LY
Ruminal pH	6.5	6.6	6.3	6.3	0.19	0.050	0.058	0.463
Temperature, °C	38.8	39.1	39.1	39.3	0.15	0.341	0.305	0.221
NH_3_-N, mg/dL	12.3	14.6	15.8	16.9	0.43	0.028	0.015	0.659
BUN, mg/dL	10.4	10.7	11.6	11.8	0.26	0.543	0.612	0.802
Total VFAs, mmol/L	92.4	102.1	95.5	105.0	1.35	0.031	0.039	0.726
VFAs, mol/100 mol								
Acetic acid (C_2_)	67.6	65.6	64.2	62.1	0.43	0.024	0.015	0.908
Propionic acid (C_3_)	23.0	24.1	25.6	28.7	0.82	0.015	0.014	0.423
Butyric acid (C_4_)	9.4	10.3	10.2	9.2	0.35	0.678	0.779	0.706
C_2_:C_3_	2.9	2.7	2.5	2.2	1.15	0.611	0.048	0.658

R:C ratio = roughage-to-concentrate ratio; LY 0 = unsupplementation of live yeast; LY 4 = supplementation live yeast at 4 g/hd/d; SEM = standard error of the mean; NH_3_-N = ammonia nitrogen; BUN = blood urea nitrogen; VFAs = volatile fatty acids.

**Table 4:** Effect of concentrate level and live yeast supplementation on microbial population in Thai native beef cattle.

Items	R:C at 60:40		R:C at 40:60		SEM	Interaction		
	LY 0	LY 4	LY 0	LY 4		R:C	LY	R:C × LY
Ruminal microbes								
Bacteria, ×10^11^ cell/ml	4.8	5.4	5.0	5.7	0.24	0.044	0.041	0.949
Protozoa, ×10^6^ cell/ml	5.0	3.9	5.6	4.2	0.41	0.843	0.039	0.536
Fungi, ×10^5^ cell/ml	2.8	3.9	3.4	4.5	0.35	0.845	0.032	0.961

R:C ratio = roughage-to-concentrate ratio; LY 0 = unsupplementation of live yeast; LY 4 = supplementation live yeast at 4 g/hd/d; SEM = standard error of the mean.

**Table 5:** Effect of concentrate level and live yeast supplementation on urinary purine derivatives (PD) and microbial protein synthesis in Thai native beef cattle.

Items	R:C at 60:40		R:C at 40:60		SEM	Interaction		
	LY 0	LY 4	LY 0	LY 4		R:C	LY	R:C × LY
Urinary purine derivatives, mmol/d								
Allantoin excretion	17.9	21.5	20.3	24.6	4.39	0.023	0.036	0.509
Allantoin absorption	49.6	56.4	53.8	62.9	2.14	0.041	0.043	0.084
MNS, gN/d	31.6	35.4	33.9	38.6	2.03	0.044	0.042	0.751
EMNS, g/kg OMDR	12.5	16.1	15.6	18.2	1.06	0.045	0.043	0.216

R:C ratio = roughage-to-concentrate ratio; LY 0 = unsupplementation of live yeast; LY 4 = supplementation live yeast at 4 g/hd/d; SEM = standard error of the mean; MNS = microbial nitrogen supply; EMNS = efficiency of microbial nitrogen synthesis; OMDR = digestible organic matter apparently fermented in the rumen.

## Data Availability

Not applicable.
